# The propagation speed of optical speckle

**DOI:** 10.1038/s41598-023-35990-z

**Published:** 2023-06-05

**Authors:** Zhenyu Wan, Murat Yessenov, Miles J. Padgett

**Affiliations:** 1 School of Physics and Astronomy, University of Glasgow, Glasgow, G12 8QQ UK; 2 CREOL, The College of Optics and Photonics, University of Central Florida, Orlando, FL 32186 USA

**Keywords:** Optical physics, Optics and photonics, Optical physics

## Abstract

That the speed of light in vacuum is constant is a cornerstone of modern physics. However, recent experiments have shown that when the light field is confined in the transverse plane, the observed propagation speed of the light is reduced. This effect is a consequence of the transverse structure which reduces the component of wavevector of the light in the direction of propagation, thereby modifying both the phase and group velocity. Here, we consider the case of optical speckle, which has a random transverse distribution and is ubiquitous with scales ranging from the microscopic to the astronomical. We numerically investigate the plane-to-plane propagation speed of the optical speckle by using the method of angular spectrum analysis. For a general diffuser with Gaussian scattering over an angular range of 5°, we calculate the slowing of the propagation speed of the optical speckle to be on the order of 1% of the free-space speed, resulting in a significantly higher temporal delay compared to the Bessel and Laguerre–Gaussian beams considered previously. Our results have implications for studying optical speckle in both laboratory and astronomical settings.

## Introduction

The speed of light is a fundamental property of light, both in terms of waves and photons. It is generally accepted that the velocity in vacuum is a constant *c*, which is one of the fundamental units of nature from which the unit of length is defined^1^. The optical physics community, however, has been fascinated by controlling and observing deviations from this constant. One well-known example is the related phenomena of slow and fast light^2–4^, where the group velocity of light pulses is modified through a material system, including atomic vapors^5^, ultracold atoms^6^, optical fibers^7–9^, photonic crystals^10^, and so on^11–14^. The basis of these effects is generally associated with the chromatic dispersion of a light pulse, which tends to spread or distort temporally as it propagates through an optical medium. An alternative mechanism to control the group velocity of light is via propagation-invariant wave packets with underlying spatiotemporal structure^15^, such as Bessel-X pulses^16^, and space–time wave packets^17, 18^. Based on these phenomena, various strategies have been proposed to realize the superluminal propagation^19–22^, and arbitrarily adjustable group velocities^23–26^ in free space. Such implementations are facilitated by space–time coupling, where the light pulses undergo spatiotemporal sculpting via tight correlation between spatial and temporal degrees of freedom^15, 18^.

In addition to these various phenomena, it has more recently been recognized that the transverse confinement of a wave or the spatial structure of a single photon will modify its propagation speed, resulting in a subluminal group velocity^27^. This modification derives from the divergence or convergence of the beam on account of the beam’s transverse structure. Such slowing of the propagation speed, induced by spatial structure, we term “structured slow light”, which can occur in the absence of any medium. For a simple example, within a hollow waveguide, the transverse modes travelling between two planes yield a group velocity less than *c*^28^. According to the theory of waveguides, the relationship between phase velocity *v*_*ϕ*_ and group velocity *v*_*g,z*_ along the waveguide appears as *v*_*ϕ*_*v*_*g,z*_ = *c*^2^^29^. This means that, considering the reduction of the axial projected wavevector *k*_*z*_ along the guide versus the fixed wavenumber *k*_0_, there is a phase velocity exceeding *c*, and it results in a reduced group velocity, where $k_{0} = {{2\pi } \mathord{\left/ {\vphantom {{2\pi } \lambda }} \right. \kern-0pt} \lambda }$ and *λ* is the optical wavelength. It should be emphasised here that this slowing is not caused by the waveguide directly but rather by the boundary conditions that the waveguide imposes on the transverse spatial structure.

It is worth noting that the slowing effect of this structured light is distinct from the local group velocity changing near the focus caused by the Gouy phase shift^30, 31^, although they are both related to the transverse spatial restrictions of the beam. The slowing of structured light persists from the near field to the far field, so the total delay during propagation is much greater than the impact of Gouy phase effect which only occurs near the focus.

In cylindrical coordinates, the free space dispersion relation takes the form $k_{z}^{2} + k_{r}^{2} = ({\omega \mathord{\left/ {\vphantom {\omega c}} \right. \kern-0pt} c})^{2}$, where $k_{r} = k\sin \theta$ and $k_{z} = k\cos \theta$ are the radial and axial components of the wavevector, *ω* is the temporal frequency and *θ* is the wavevector angle with respect to the beam axis. Taking the case of Bessel-type beams as an example, there are three alternative ways of generating polychromatic beams with transverse localization that lead to distinct propagation properties. Firstly, Bessel-X waves with frequency-independent propagation angle $\theta$ that possess diffraction-free and dispersion-free propagation at superluminal group velocities^20^, secondly, propagation-invariant 3D space–time wave packets with a parabolic space–time coupling $k_{r} \propto \sqrt {\left| {\omega - \omega_{0} } \right|}$ that leads to arbitrary group velocities in free space (ω_0_ is the central frequency)^32^, and finally Bessel–Gauss pulsed beams with frequency-independent radial wavevector *k*_*r*_ traveling at subluminal group velocities in free space^33, 34^. Bessel-X waves generated via axicon and 3D space–time wave packets retain an X-shaped spatio-temporal profile due to the space–time coupling along with the Bessel-type transverse profile.

To illustrate the differences, in the case of Bessel-X waves, the space–time coupling takes the form of ${{k_{z} } \mathord{\left/ {\vphantom {{k_{z} } k}} \right. \kern-0pt} k} = \cos \alpha$, where *α* is axicon angle, leading to a superluminal value for both the phase velocity and group velocity, i.e., ${{v_{\phi } = v_{g} = c} \mathord{\left/ {\vphantom {{v_{\phi } = v_{g} = c} {\cos \alpha }}} \right. \kern-0pt} {\cos \alpha }}$. On the contrary, Bessel–Gauss pulsed beams synthesized using an annular slit or an equivalent diffractive element are endowed with one spatial frequency *k*_*r*_ for all temporal frequencies *ω*, leading to a dispersive propagation at subluminal group velocities in free space^34^. Due to the fixed spatial spectrum *k*_*r*_ over the whole spectral bandwidth in the latter case, at the quasi-monochromatic limit, one can consider these beams as spatially structured fields without regarding their spatio-temporal correlation^27^. In the paraxial regime, unlike space–time wave packets whose group velocity is tunable over a broad range of values^32^, the variation of the group velocities of Bessel-X waves and Bessel–Gauss pulsed beams from *c* is limited by the numerical aperture (NA) of the system^19, 20^.

Looking beyond Bessel beams, more generally, when considering the group velocity or similar metrics for propagation speed of a finite length pulse it is important to recognise that any finite length pulse has a spread of *k*_0_ values, albeit potentially very small. In this regard it is essential that the derivatives of the various component of *k* with respect to *k*_0_ are examined. When generating structured light beam there are two different approaches that should be considered. The first of these approaches is when using a refractive or reflective optic where, ignoring dispersion, the transverse components of *k*, i.e., *k*_*x*_ and *k*_*y*_ scale linearly with *k*_0_. The second of these approaches is when using a diffractive optic where the transverse component of *k* are independent of *k*_0_. In our case we are considering the second of these two approaches where the diffractive is implemented using a spatial light modulator (SLM), in its off-axis mode. Throughout the rest of this work we are assuming the case of where the transverse component of *k* are independent of *k*_0_.

In recent years, both theoretical analyses and experimental demonstrations have been carried out for revealing the effect of structured slow light as applied to Bessel beams, focused beams^27, 35^, Laguerre–Gaussian (LG) beams^36, 37^, and the intrinsic effect of orbital angular momentum (OAM)^38^. For example, the experimentally observed slowing, or corresponding group delay, in Giovannini’s experiments^27^ is around one part in 10^5^ compared with the reference values. It is restricted by a small spatial divergence of the beams, which corresponds to the skew trajectories of optical rays in the geometrical optics^39^. This slowing of a structured light beam was shown to scale with the square of its divergence, expressed quantitatively as $\theta = {{k_{r} } \mathord{\left/ {\vphantom {{k_{r} } {k_{0} }}} \right. \kern-0pt} {k_{0} }}$ within the small-angle approximation^27^. The maximum divergence of the light is limited by the numerical aperture of the supporting optical system which is defined as the ratio between the limiting aperture and the distance from that aperture. To calculate the time delay associated with this reduction in the propagation speed one needs also to account for the distance over which the propagation occurs. Hence for structured light beams to be produced and detected with a fixed aperture, the combination of the scaling of the slowing together with the propagation distance means that the maximum temporal delay scales inversely with the propagation distance, i.e., it is a short-range effect. Taking the Bessel beam as an example, for a finite radius, a longer diffraction-free propagation distance is maintained by a smaller cone angle^40^, which reduces the slowing effect. In this present work we consider not a specifically structured beam but instead the general case of random optical speckle, which can be created over a very large field of view and with long propagation distances allowing the possibility of significant temporal delays.

## Methods and results

**Figure 1:**
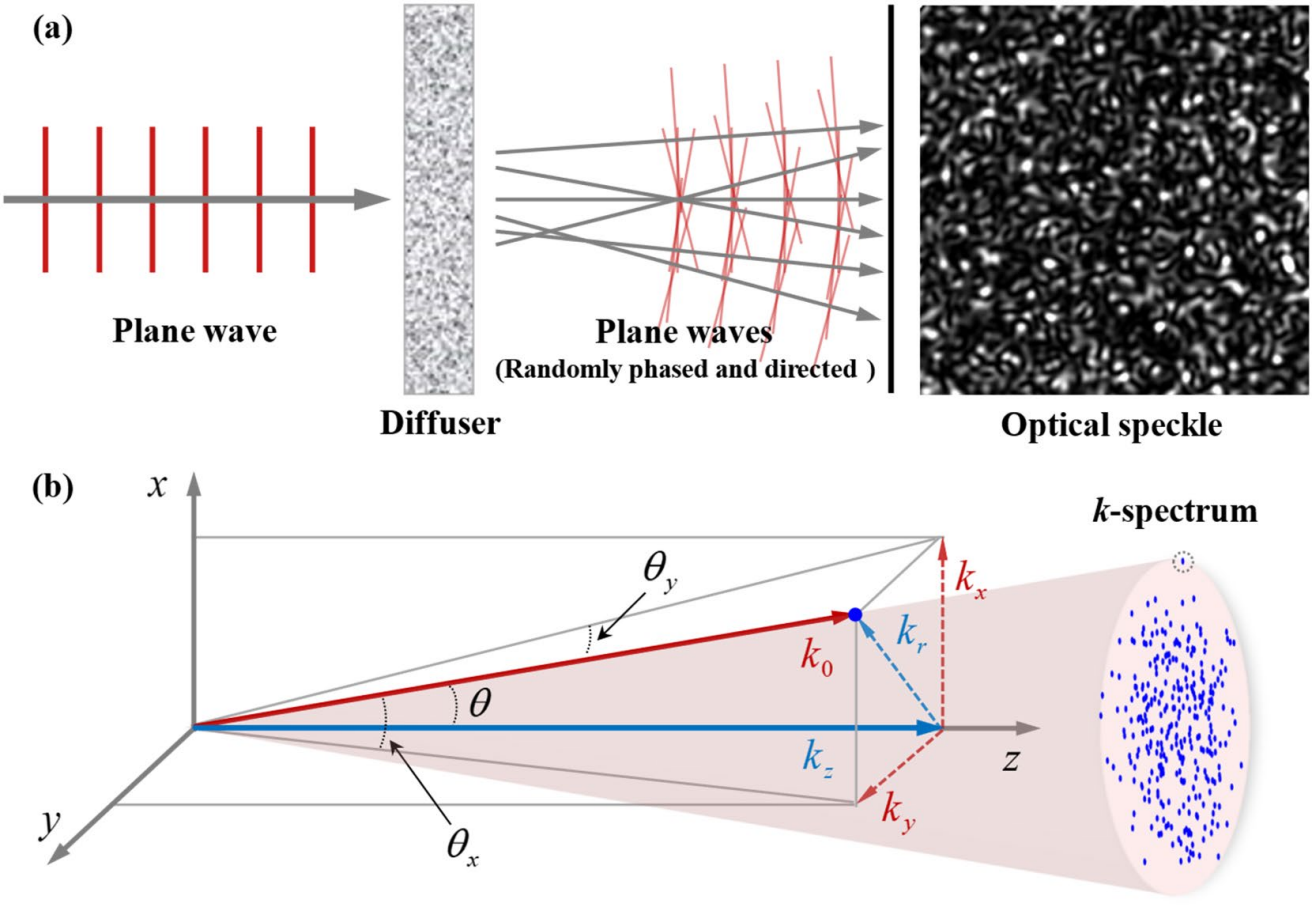
Optical speckle in free space and *k*-space. (**a**) Superposition between a sufficiently large set of randomly phased and directed plane waves is an approximation to the optical speckle created by scattering a laser beam from a diffuser. (**b**) *k*-spectrum of optical speckle and the projection of one of the points in direction space of wave vectors.

Optical speckle arises from the interference between random distributions of plane wave components, such as generated by light scattering from rough surfaces or propagating through turbid diffusers^41^. For example, when a laser is incident on an object such as ground glass or scattering screen, the transmitted or reflected light would be observed with fine-scale granular pattern. According to the Huygens-Fresnel principle, the optical speckle resulting from scattering coherent light can be considered as the interference caused by different scattering points that act as individual new nearly-spherical wave sources. Since the solid angle subtended by the detecting system is sufficiently small, each spherical wave in the volume of space around the viewing aperture is approximated by a plane wave. Hence the plane-wave approximation is widely used to simulate the optical speckle mathematically^42, 43^. In this work we model the optical speckle as a superposition of a large number of plane waves with random phases and directions, as shown in Fig. 1a. The intensity pattern of the speckle has a grainy appearance, where the bright spots and dark specks arise from the constructive and destructive interference respectively. In particular, the centre of each dark speck is a phase singularity, and in 3 dimensions these dark filaments thread themselves through the speckle field creating highly complicated networks of vortex lines and loops^44–46^. Intuitively, the angular spectrum of light field can be mapped to direction space of wave vectors (*k*-space), i.e., with amplitude correspondence to the *k*-spectrum, where each point represents a plane wave, to which is assigned random transverse projected components (*k*_*x*_ and *k*_*y*_), as shown in Fig. 1b. The corresponding nonzero radial component $k_{r} = \sqrt {k_{x}^{2} + k_{y}^{2} }$ produces a modification of average axial component $\left\langle {k_{z} } \right\rangle = \sqrt {k_{0}^{2} - \left\langle {k_{r}^{2} } \right\rangle }$, where $\left\langle {...} \right\rangle$ denotes the statistic expectation over the *k*-spectrum.

To characterize the propagation speed of optical speckle, we introduce the phase and group velocities which are averaged across all the wave components velocity which we have previously shown corresponds to the time the light or photons take to travel from plane to plane. Different from the conventional definition of group velocity^47^, the spatially average group velocity refers to the traveling energy envelope of a group of plane waves with a small spread in directions (i.e., spatial components in *k*-spectrum) rather than in frequencies or wavenumbers (i.e., temporal components in frequency spectrum). The spatially averaged phase velocity is then given as $v_{\phi } = c \cdot {{k_{0} } \mathord{\left/ {\vphantom {{k_{0} } {\left\langle {k_{z} } \right\rangle }}} \right. \kern-0pt} {\left\langle {k_{z} } \right\rangle }}$ by the average value of *k*_*z*_. For a structured beam in free-space it seems sensible that the average group velocity and phase velocity have the same relation as in the theory of hollow waveguides, i.e., $v_{\phi } v_{g,z} = c^{2}$. The condition is best satisfied when we assume that the radial projection of wave vector *k*_*r*_ in optical speckle analyzed here is independent on its angular frequency *ω*. The resultant spatially average group velocity along *z* is thus given as

1 $$v_{g,z} = c \cdot \sqrt {1 - {{\left\langle {k_{r}^{2} } \right\rangle } \mathord{\left/ {\vphantom {{\left\langle {k_{r}^{2} } \right\rangle } {k_{0}^{2} }}} \right. \kern-0pt} {k_{0}^{2} }}} ,$$

meaning that structured beams with a nonzero expectation value of $k_{r}^{2}$, of which optical speckle is one example, will experience a reduced propagation speed, i.e., *v*_*g,z*_ < *c*.

We emphasize that the optical field considered here is quasi-chromatic, i.e., the frequencies of the wave group are clustered in a very narrow region around the main frequency. The field endowed with fixed ${k}_{r}$ still experiences group velocity dispersion (GVD) when the input beam is pulsed. This is another distinction between the effect of structured slow light and the group velocity control with space–time wave packets, which results in dispersion-free propagation^48, 49^. However, in the structured slow light the amount of GVD is insignificant compared with the differentiable group delay $\tau_{DGD} = L\left| {\frac{1}{c} - \frac{1}{{v_{g} }}} \right|$ acquired by this pulse, where *L* is the axial propagation distance. It can be shown that for the pulse of spectral bandwidth of Δ*ω* and spatial wavevector *k*_*r*_ the ratio of pulse broadening Δ*τ* to the differentiable group delay $\tau_{DGD}$ is proportional to $\frac{\Delta \omega }{{\omega_{0} }}$, which is at the quasi-monochromatic regime is negligible, i.e. $\frac{\Delta \tau }{{\tau_{DGD} }} \sim \frac{\Delta \omega }{{\omega_{0} }} \ll 1$^49, 50^.

As introduced earlier, to experimentally generate an optical speckle with *k*_*r*_ components that are independent of *k*_0_ requires diffractive elements, e.g., superposed grating patterns uploaded on SLM. For a single randomized plane wave produced by a hologram of grating pattern with fringe separation *d*, the resultant transverse components *k*_*x*_ (*k*_*y*_) is 2π/*d*_*x*_ (2π/*d*_*y*_), and *k*_*r*_ is independent of wavelength. Each plane-wave hologram is assigned with three individual variables: polar angle, azimuthal angle and phase offset, where the polar angles are distributed with a Gaussian profile, and both azimuthal angles and phase offsets are uniform noise. The resulting phase hologram uploaded on SLM comprises the wavevectors of optical speckle by combining the grating patterns.

**Figure 2:**
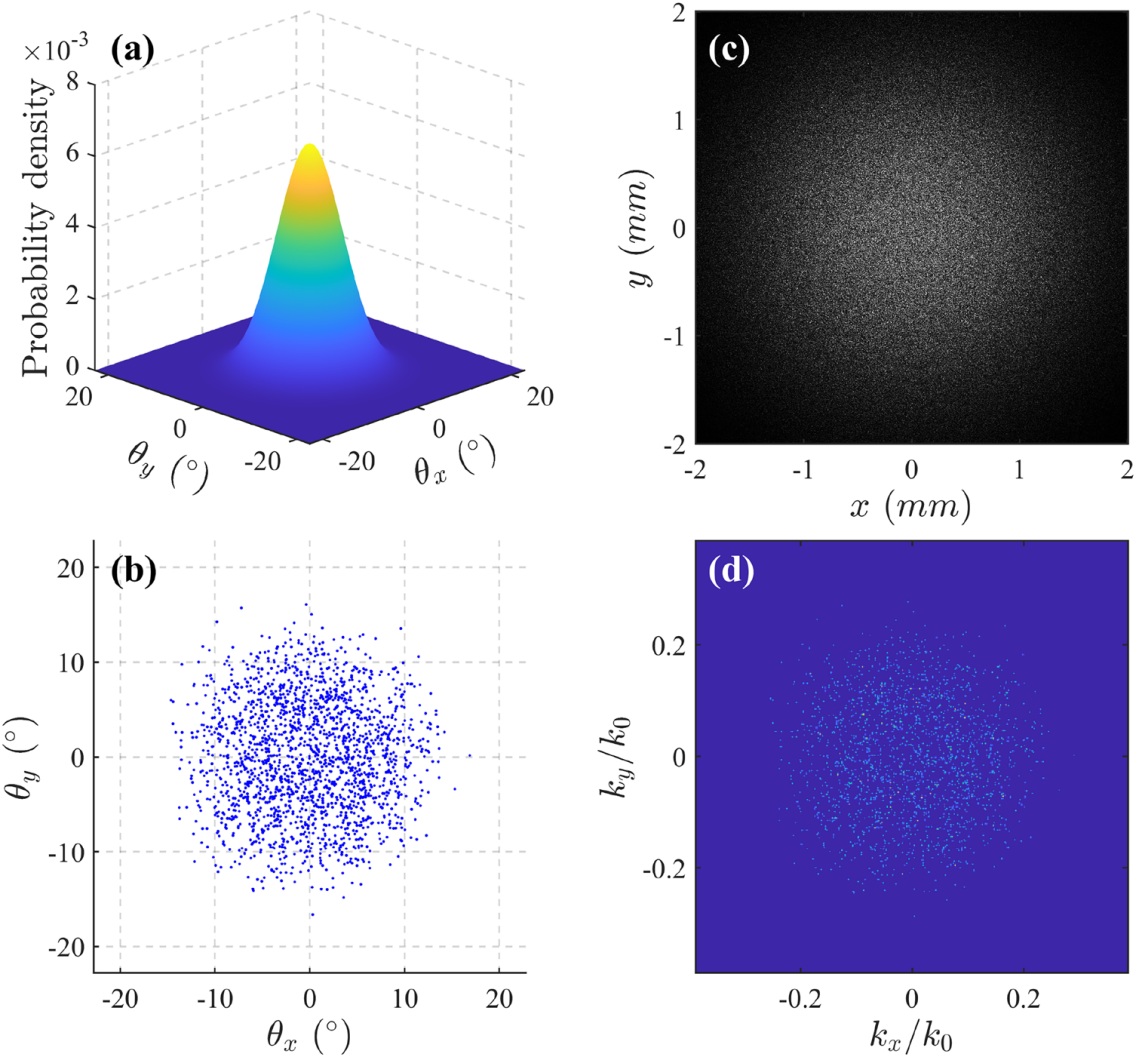
An example of numerically generating optical speckle. (**a**) Gaussian probability density distribution of tilted directions in *k*-space. (**b**) Direction points with Gaussian density of standard deviation of 5°. (**c**) Intensity profile of optical speckle created by the interference between Gaussian random waves with directions as (**b**). (**d**) Calculated k-spectrum of speckle field by 2D Fourier transform of its complex amplitude.

To model such optical speckle numerically, we define a finite two-dimensional grid in transverse *k*-space, where each point describes a plane wave tilted by *θ*_*x*_ and *θ*_*y*_ with respect to the propagation axis. According to the central limit theorem^51^, the superpositions of infinitely many waves tend to Gaussian random functions^52^. The ensembles of plane harmonics are asymptotically Gaussian, which means that the probability density distribution of each tilted direction (*θ*_*x*_ and *θ*_*y*_) follows a 2D Gaussian distribution, as shown in Fig. 2a. Our simulation for optical speckle is based on a superposition of 2000 plane waves randomly distributed in direction and phase, and each of which has a Gaussian amplitude in profile. Their distribution in *k*-space is subject to a Gaussian density distribution, characterized by a divergence of sin *σ*_*θ*_ in free space, where *σ*_*θ*_ is the standard deviation of the tilted angles of the wave vectors. A typical example for *σ*_*θ*_ = 5° is calculated in Fig. 2b. The resultant intensity profile of the optical speckle in the far field is shown in Fig. 2c. By performing 2D Fourier transform for the complex amplitude of the speckle field, its *k*-spectrum is obtained as shown in Fig. 2d, where the coordinates are divided by the initial wavenumber *k*_0_. It can be seen that the *k*-spectrum of optical speckle has a 2D Gaussian density envelope, which depends on the distribution of tilted directions of wave vectors in Fig. 2b. More importantly, in the paraxial regime, the effect of light propagating over *z* in free space is simply a phase change in the components of its angular spectrum, and then since the *k*-spectrum is equivalent to the modulus of angular spectrum mathematically, the *k*-spectrum of optical speckle is propagation-invariant, which means that its slowing persists over arbitrarily long ranges.

Optical speckle is usually characterized by its lateral size, which refers to the lowest length scale at which beam is correlated^53^. Particularly, for a fully developed speckle field created by a scattering surface, the size of speckle increases with the distance from the surface to observation plane^54, 55^. From the perspective of plane wave interference, the larger the tilted angle, a greater the transverse phase varying gradient and then the denser the interference fringes. In a Fourier sense, statistical properties with high complexity in real space correspond to an expanded angular spectrum. This means that the *k*-spectrum range of speckles is negatively correlated with speckle size.

**Figure 3:**
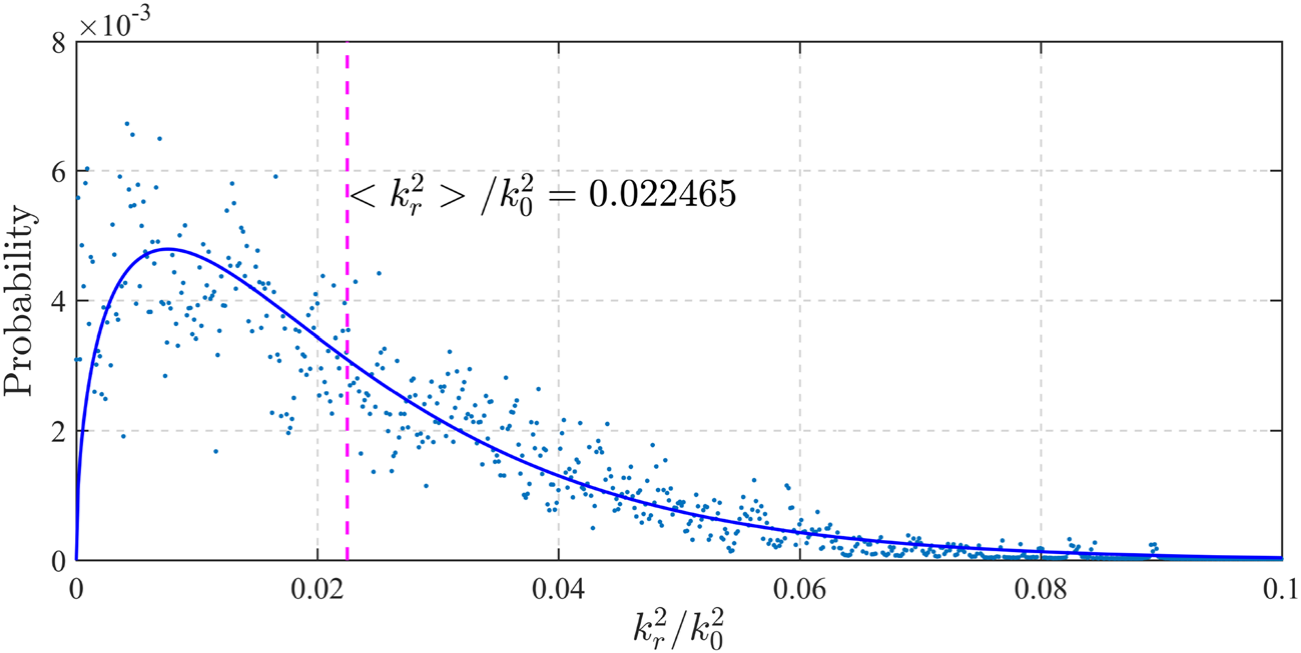
Statistical distribution of tilted components in optical speckle. The discrete points represent the probability distribution of the *k*-spectrum components calculated along the scales of radial proportion square. The solid curve is the theoretical probability density distribution of radial proportion square from an ideal continuous Gaussian angular spectrum.

To evaluate the degree of slowing for this numerically creating optical speckle, we divide the *k*-spectrum in Fig. 2d radially according to the evenly equidistant 1000 scales on the established ${{k_{r}^{2} } \mathord{\left/ {\vphantom {{k_{r}^{2} } {k_{0}^{2} }}} \right. \kern-0pt} {k_{0}^{2} }}$ axis. By summing and normalizing all the amplitudes with the individual ring regions divided from *k*-spectrum, each ring is calculated as a value point with global normalized probability along the ${{k_{r}^{2} } \mathord{\left/ {\vphantom {{k_{r}^{2} } {k_{0}^{2} }}} \right. \kern-0pt} {k_{0}^{2} }}$ axis, as shown in Fig. 3. Physically, each discrete point represents the probability of a plane wave that appears within a ${{\Delta k_{r}^{2} } \mathord{\left/ {\vphantom {{\Delta k_{r}^{2} } {k_{0}^{2} }}} \right. \kern-0pt} {k_{0}^{2} }}$ ring region of *k*-space, where $\Delta k_{r}^{2}$ is the division value on axis. In this case (*σ*_*θ*_ = 5°), the value ${{\left\langle {k_{r}^{2} } \right\rangle } \mathord{\left/ {\vphantom {{\left\langle {k_{r}^{2} } \right\rangle } {k_{0}^{2} }}} \right. \kern-0pt} {k_{0}^{2} }}$ is calculated as 0.022465, and then the spatially average group velocity of such optical speckle is calculated by Eq. (1) as $v_{g,z} \approx 0.9887c$. This means that the propagation speed of an optical speckle with the Gaussian divergence with standard deviation of 5° corresponds to a slowing of 1.13% in free space.

In addition to the discrete sampling of the *k*-spectrum of optical speckle as an example, a continuous probability density distribution of radial proportion square ${{k_{r}^{2} } \mathord{\left/ {\vphantom {{k_{r}^{2} } {k_{0}^{2} }}} \right. \kern-0pt} {k_{0}^{2} }}$ can be deduced from the Gaussian angular spectrum mathematically as

2 $$p\left( {{{k_{r}^{2} } \mathord{\left/ {\vphantom {{k_{r}^{2} } {k_{0}^{2} }}} \right. \kern-0pt} {k_{0}^{2} }}} \right) = {{\sqrt {2\pi \cdot {{k_{r}^{2} } \mathord{\left/ {\vphantom {{k_{r}^{2} } {k_{0}^{2} }}} \right. \kern-0pt} {k_{0}^{2} }}} } \mathord{\left/ {\vphantom {{\sqrt {2\pi \cdot {{k_{r}^{2} } \mathord{\left/ {\vphantom {{k_{r}^{2} } {k_{0}^{2} }}} \right. \kern-0pt} {k_{0}^{2} }}} } {\sin \sigma_{\theta } }}} \right. \kern-0pt} {\sin \sigma_{\theta } }} \cdot \exp \left( { - \frac{{{{k_{r}^{2} } \mathord{\left/ {\vphantom {{k_{r}^{2} } {k_{0}^{2} }}} \right. \kern-0pt} {k_{0}^{2} }}}}{{2\sin^{2} \sigma_{\theta } }}} \right),$$

where sin *σ*_*θ*_ again refers to the divergence of optical speckle. Figure 3 shows a good fit between the theoretical curve of Eq. (2) and the sampling points from a typical k-spectrum in Fig. 2d.

**Figure 4:**
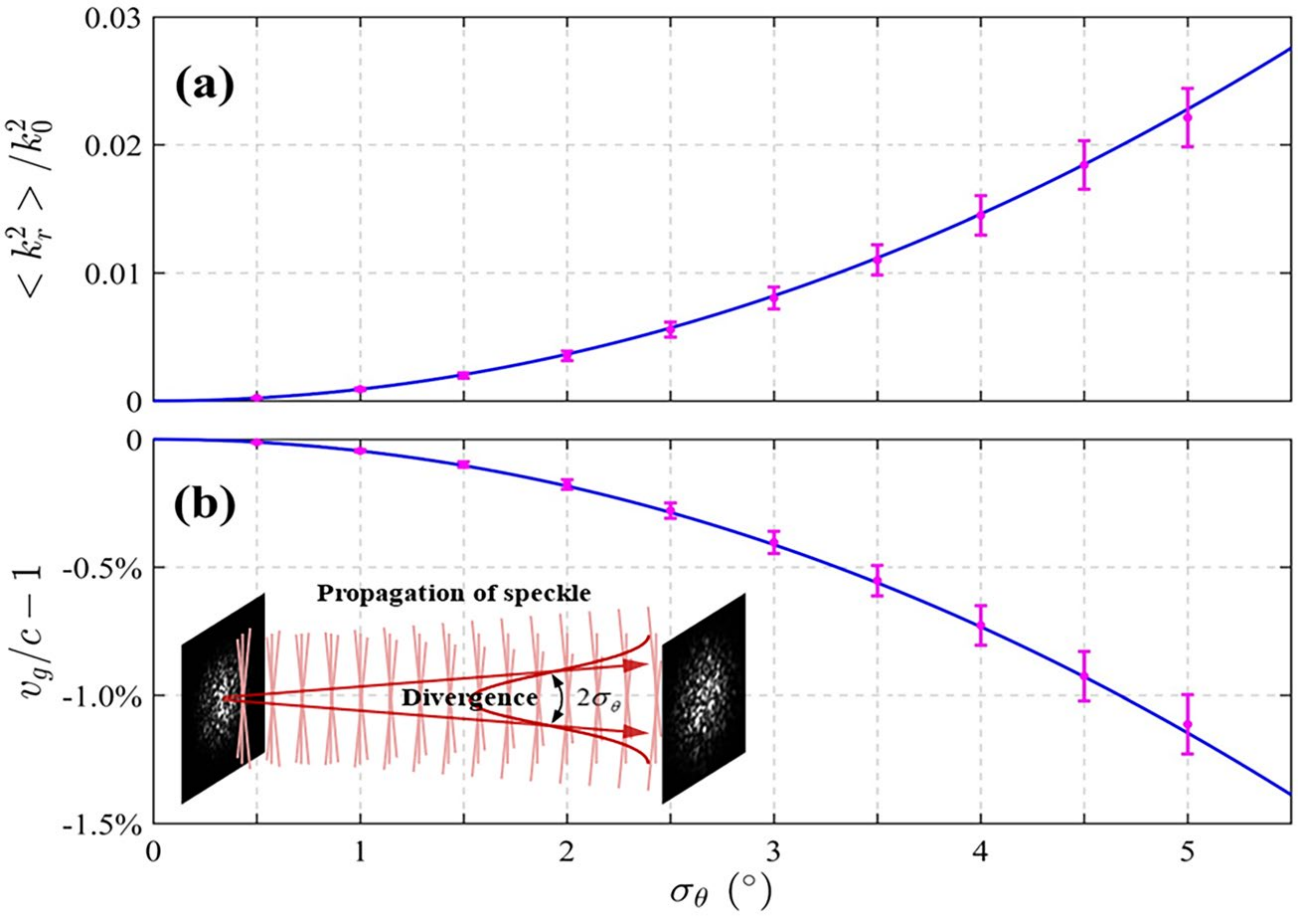
Numerically quantifying slowing effect of optical speckle. (**a**) Expectation values of radial proportion square and (**b**) degree of slowing under different divergence of optical speckle. The inset is a schematic of divergence of optical speckle where *σ*_*θ*_ is the half spreading angle that describes the tilted plane-wave components.

We perform a numerical analysis for the relation between slowing effect as a function of the divergence of the optical speckle. In particular, the divergence refers to the spreading angle, which describes the standard deviation of the tilted angles of the wave vectors, as shown in the inset of Fig. 4. By gradually adjusting *σ*_*θ*_ from 0.5° to 5° at 0.5° intervals, we calculate the value ${{\left\langle {k_{r}^{2} } \right\rangle } \mathord{\left/ {\vphantom {{\left\langle {k_{r}^{2} } \right\rangle } {k_{0}^{2} }}} \right. \kern-0pt} {k_{0}^{2} }}$ and corresponding slowing, as plotted in Fig. 4. For each case, the differences in the generation of Gaussian distributed random numbers within a diverging range would result in variation of the slowing predicted, and hence the error bar is derived from performing the calculation 8 times using the method of Fig. 3. As anticipated, the predicted slowing effect becomes greater as the divergence increases.

Beyond the numerical simulations described above, the theoretical expression of slowing effect also deduced. According to the probability density distribution of radial proportion square ${{k_{r}^{2} } \mathord{\left/ {\vphantom {{k_{r}^{2} } {k_{0}^{2} }}} \right. \kern-0pt} {k_{0}^{2} }}$ in Eq. (2), its expected value is calculated as

3 $${{\left\langle {k_{r}^{2} } \right\rangle } \mathord{\left/ {\vphantom {{\left\langle {k_{r}^{2} } \right\rangle } {k_{0}^{2} }}} \right. \kern-0pt} {k_{0}^{2} }} = \frac{{\int_{0}^{\infty } {p\left( {{{k_{r}^{2} } \mathord{\left/ {\vphantom {{k_{r}^{2} } {k_{0}^{2} }}} \right. \kern-0pt} {k_{0}^{2} }}} \right)} \cdot {{k_{r}^{2} } \mathord{\left/ {\vphantom {{k_{r}^{2} } {k_{0}^{2} }}} \right. \kern-0pt} {k_{0}^{2} }} \cdot d\left( {{{k_{r}^{2} } \mathord{\left/ {\vphantom {{k_{r}^{2} } {k_{0}^{2} }}} \right. \kern-0pt} {k_{0}^{2} }}} \right)}}{{\int_{0}^{\infty } {p\left( {{{k_{r}^{2} } \mathord{\left/ {\vphantom {{k_{r}^{2} } {k_{0}^{2} }}} \right. \kern-0pt} {k_{0}^{2} }}} \right)} \cdot d\left( {{{k_{r}^{2} } \mathord{\left/ {\vphantom {{k_{r}^{2} } {k_{0}^{2} }}} \right. \kern-0pt} {k_{0}^{2} }}} \right)}} = 3\sin^{2} \sigma_{\theta } ,$$

where the infinite of upper limit in the integral is only mathematically meaningful for its normalization among the whole space, while more strictly in physics, the upper limit should be 1 since *k*_*r*_ < *k*_0_. Clearly, ${{\left\langle {k_{r}^{2} } \right\rangle } \mathord{\left/ {\vphantom {{\left\langle {k_{r}^{2} } \right\rangle } {k_{0}^{2} }}} \right. \kern-0pt} {k_{0}^{2} }}$ is proportional to square of the divergence of optical speckle, see the solid curve in Fig. 4a. Using Eq. (1), for small angles *σ*_*θ*_, the degree of slowing of optical speckle is theoretically calculated as

4 $${{v_{g,z} } \mathord{\left/ {\vphantom {{v_{g,z} } c}} \right. \kern-0pt} c} - 1 = \sqrt {1 - 3\sin^{2} \sigma_{\theta } } - 1.$$

Figure 4b indicates the agreement between the theoretical curve and the mean values of each result calculated by discrete statistical method. Note that Eq. (4) is only applicable for the low-NA case to ensure paraxial approximation. Significantly, the slowing of the optical speckle can reach of order 1% even with a small beam divergence. Over the range of several meters, the temporal delay of optical speckle is thus predicted to be enhanced by three orders of magnitude for the same traveling distance compared to the previously measured Bessel or focused beams^27^.

**Figure 5:**
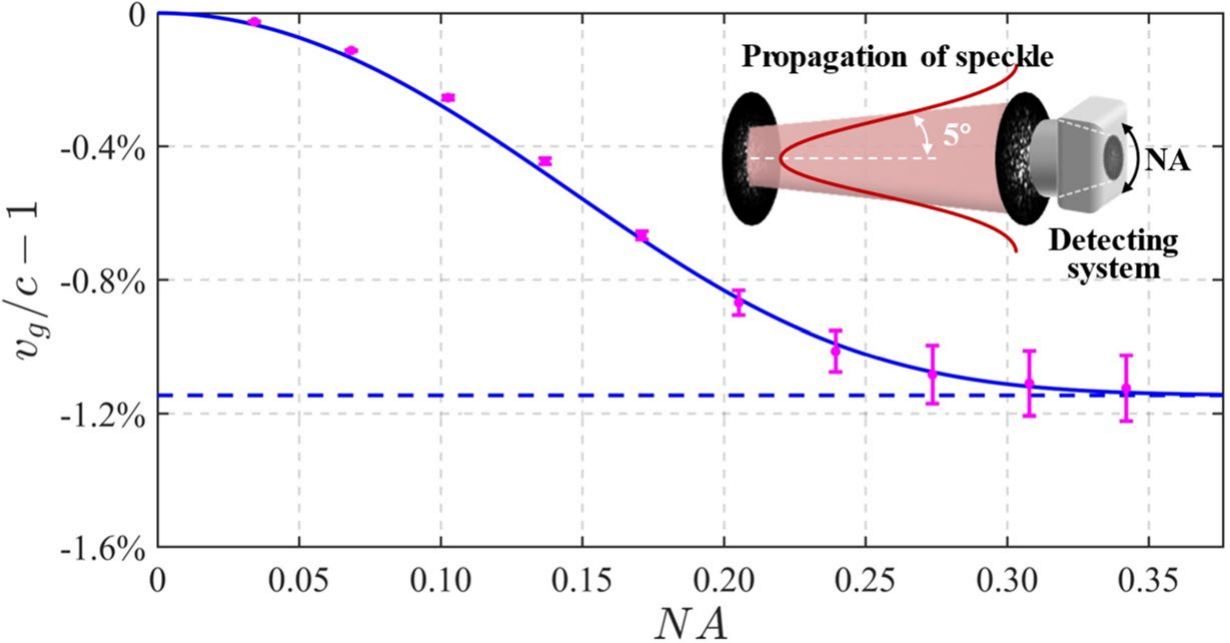
Restrictions of practical system on slowing effect of optical speckle where the itself speckle has a divergence of 5° and the detector has a limiting NA. The degree of slowing calculated under different numerical apertures (NA) of detecting system. The inset is a schematic of a detecting system for observing the plane-to-plane propagation of optical speckle.

To anticipate the observable slowing in a practical detecting system, we consider the role that the aperture of the detector plays. The NA is a restriction on *k*-space when the optical speckle is observed by a detector or our eyes, as shown in the inset of Fig. 5. When considering the restriction on complete spatial harmonics collecting of optical speckle by the detecting system, the upper limit of the integral in Eq. (3) is replaced by NA^2^ from infinite. In the initialization settings of calculation here, the beam waist of Gaussian-distributed intensity profile of the optical speckle is set to 2 mm, and its half spreading angle is set to 5°. Figure 5 shows the calculated degree of slowing under different NA, where the dashed line predicted by Eq. (4) refers to the ideal case without restriction of NA, and the solid curve is predicted by modified Eq. (3), and the data points are obtained with 8 calculations by filtering the complex amplitude of speckle field in *k*-spectrum. Since the NA is a restriction of maximum range of angular spectrum, the angular spectrum outside this range is filtered out, which is analogue to a low-pass filtering while the higher components in *k*-spectrum give a greater slowing. This means that reducing the NA of the detection system will obviously reduce the corresponding slowing effect, which is seen in Fig. 5. In contrast, a beam aperture, i.e., a transverse restriction to the propagation of light in real space, would not impact the slowing effect drastically since whole spatial harmonics can pass the aperture, but the restriction of beam aperture would reduce the resolution of the *k*-spectrum of field due to the correspondence of the maximum of beam size to the minimum of *k*-space. Note that the structured slowing effect analyzed in this work is a global property. However, when one observes the local grain of optical speckle, the structured slowing effect is preserved even within a small region of interest, as all transverse *k*_*r*_ could contribute to the light behavior in this region.

## Discussions and conclusions

In conclusion we have reasoned that the slowing of structured light beams in free space^27^ extend beyond the focused-Gaussian and Bessel beams considered in that work to include random structuring such as optical speckle. In all cases, the slowing arises from a non-zero component of the transverse wavevector which reduces the axial component of the wavevector below the free-space, plane-wave value. As in the case of a hollow waveguide, this reduction increases the phase velocity along the optical axis above *c*, which in turn reduces the group velocity below *c*. Since the angular distribution of wavevectors describing a beam does not change upon propagation in free-space, this slowing is not restricted to the vicinity of the focus, rather it persists into the far-field. The scale of the slowing depends upon the limiting numerical aperture associated with the generation, transmission and detection, whichever is the lower.

In our analysis we have restricted ourselves to Cartesian or radial coordinates which are suited to optical configuration with modest numerical aperture. However, we note that the slowing predicted scales quadratically with the numerical aperture an although outside of the scope of this work, or indeed any experiments to date, it raises a question as to what the equivalent effect might be for scenarios where the speckle subtends over a large solid angle such as 4Pi-confocal microscopy^56^.

Another intriguing example of high numerical aperture systems exhibiting speckle is the cosmic microwave background (CMB) anisotropies. This has many parallels with the formation of speckles, where the microwave photons stream freely from the surface of last scattering to the observer and the intrinsic anisotropy of which is recognized as the small temperature fluctuations imprinted on the surface of last scattering^57^. According to the measured data of power spectrum^58^, the temperature fluctuations of CMB show a function of angular scale. Could it be that the CMB patterns experience similar slowing effects as the high-NA speckle and more that the CMB patterns seen from different angular scales may have different arrival times?

Finally, for both low and high NA, it is interesting to reflect on the fact that the spatial encoding of data onto a light beam’s transverse structure necessitates a transverse component to the wavevector and hence an associated slowing. Such a slowing therefore seems to be an inescapable consequence of the spatial structure as expressed in terms of light’s spatial information content or entropy.

These considerations are a subject of our ongoing studies.

## Data Availability

The MATLAB codes for the full set of results are available online in University of Glasgow Library Data Repository (http://dx.doi.org/10.5525/gla.researchdata.1414).
